# Effect of lactation feeder design on sow and litter performance, feeder cleaning criteria, and economic return

**DOI:** 10.1093/tas/txae146

**Published:** 2024-10-07

**Authors:** Rafe Q Royall, Kyle F Coble, Karley R Stephens, Mike D Tokach, Jason C Woodworth, Joel M DeRouchey, Robert D Goodband, Jordan T Gebhardt, Jimmy Karl, Paul J Corns, Tag Bradley

**Affiliations:** Department of Animal Sciences and Industry, College of Agriculture, Kansas State University, Manhattan, KS 66506-0201, USA; JBS Live Pork, Greeley, CO 80634, USA; Department of Agricultural Education and Communications, Davis College of Agricultural Sciences and Natural Resources, Texas Tech University, Lubbock, TX 79409-2131, USA; Department of Animal Sciences and Industry, College of Agriculture, Kansas State University, Manhattan, KS 66506-0201, USA; Department of Animal Sciences and Industry, College of Agriculture, Kansas State University, Manhattan, KS 66506-0201, USA; Department of Animal Sciences and Industry, College of Agriculture, Kansas State University, Manhattan, KS 66506-0201, USA; Department of Animal Sciences and Industry, College of Agriculture, Kansas State University, Manhattan, KS 66506-0201, USA; Department of Diagnostic Medicine/Pathobiology, College of Veterinary Medicine, Kansas State University, Manhattan, KS 66506-0201, USA; JBS Live Pork, Greeley, CO 80634, USA; JBS Live Pork, Greeley, CO 80634, USA; JBS Live Pork, Greeley, CO 80634, USA

**Keywords:** feed intake, lactation feeder, litter performance, sow, wet–dry feeder

## Abstract

A total of 557 mixed parity sows (PIC 1050) were used to evaluate the effect of lactation feeder design on sow farrowing performance, litter growth performance, feeder cleaning criteria, and economics. The experiment was conducted during the summer of 2023 at a commercial sow farm located in northwest Texas. The study used two sequential farrowing groups with approximately 279 sows per group. On approximately days 112 to 114 of gestation, sows were moved to the farrowing house and randomly allotted to one of three feeder types based on parity and caliper score. Feeder types consisted of 1) a dry feeder with a nipple drinker located next to the feeder, 2) a wet–dry feeder with a divider to separate feed and water, or 3) a wet–dry feeder without a divider. The three feeder types were used in one of every three stalls with the same sequence from the front to the end of all rooms to balance for environmental effects. Sows were weighed before entering the farrowing house and at weaning. Sows were provided approximately 1.81 kg per day of a common lactation diet prefarrowing, and after farrowing, sows were provided ad libitum access to lactation feed. There was no evidence of a difference in sow weight at entry or weaning, overall BW change, caliper score at entry or weaning, total litter weight or individual pig weight at birth, total pigs born, or percentage of pigs born alive. However, sows fed with the dry lactation feeder had decreased (*P* < 0.05) total daily feed disappearance and average daily feed disappearance compared to either wet–dry feeder design. There was no evidence of difference for litter or pig weaning weight, or litter average daily gain. As a result, litter feed efficiency was improved (*P* < 0.05) for sows fed via the dry feeder compared to either wet–dry feeder. For feeder cleaning criteria, dry feeders had increased (*P* < 0.05) washing time and washing cost compared to either wet–dry feeder design. In addition, sows fed via the dry feeder had decreased (*P* < 0.05) total lactation feed cost and feed cost per piglet weaned compared to either wet–dry feeder design. In summary, using the wet–dry feeder design in this study with or without a divider separating the feed from the water increased feed disappearance with no effects on sow and litter performance compared to dry feeders, thus worsening litter feed efficiency and increasing feed cost per sow and litter.

## Teaser

Lactation feeder design can affect sow feed intake. Compared to a dry feeder, the wet–dry feeders evaluated increased feed disappearance but had no effect on sow or litter performance.

## INTRODUCTION

Maximizing feed intake during lactation is crucial to promote high levels of milk production for litter growth while simultaneously minimizing body reserve mobilization, thus improving sow longevity and subsequent reproductive performance (Patterson et al., 2011; Tokach et al., 2019). Multiple factors can affect sow feed intake, including genetics, litter size, and environmental conditions (Peng et al., 2007; Tokach et al., 2019; Bjerg et al., 2020). One factor that has received renewed attention in recent years is lactation feeder type and design. While there are numerous types of lactation feeders on the market, a good feeder design can help improve sow feed intake while reducing feed wastage (Rao et al., 2023). In growing-finishing pigs, wet–dry feeders that provide the pig with access to feed and water in the same location have been shown to increase average daily feed intake (**ADFI**) and average daily gain (**ADG**) when compared to pigs fed using a conventional dry feeder (Bergstrom et al., 2012a, 2014; Greiner et al., 2022). However, there is limited data on the effects of using a wet–dry feeder during lactation. The existing literature has observed increased lactation feed disappearance for sows fed via a wet–dry feeder compared to a dry feeder, but these trials were not conducted using modern ad libitum feeders (O’Grady et al., 1978; Pettigrew et al., 1985; Peng et al., 2007). Our hypothesis was that because wet–dry feeders increase ADFI of finishing pigs compared to those fed with a dry feeder, the same might be true for lactating sows. Therefore, the objective of this experiment was to compare farrowing performance, litter growth performance, and feeder cleaning criteria of sows fed with a dry feeder vs. two wet–dry feeder designs.

## MATERIALS AND METHODS

### General

The Kansas State University Institutional Animal Care and Use Committee approved the protocol used in this experiment (IACUC #4535). The experiment was conducted at a commercial sow farm located in northwest Texas. There were 72 stalls per room and a total of four farrowing rooms (288 stalls; 96 stalls per lactation feeder treatment) used for each group. The trial was conducted in two sequential farrowing groups for a total of 576 sows (PIC 1050, Hendersonville, TN) enrolled on the test. Farrowing crates equipped with the dry feeder was also equipped with 1 nipple waterer placed at shoulder height approximately 40 cm from the feeder, while farrowing crates equipped with either wet–dry feeder did not have an additional water source for the sow. All crates were equipped with a nipple waterer at the base of the farrowing crate for piglets. The first group of sows farrowed between June 7 and June 17, 2023, and pigs were weaned between June 29 and July 4, 2023. The second group of sows farrowed between July 4 and July 13, 2023, and pigs were weaned between July 27 and August 1, 2023.

### Animals and Treatments

**Figure 1.**
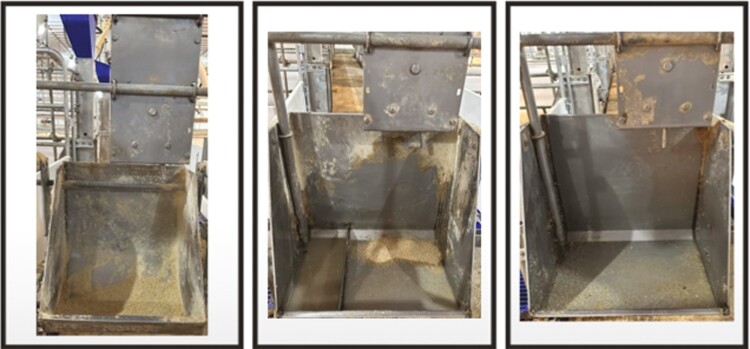
Sow lactation feeders. Dry feeder, wet–dry feeder with divider, and wet–dry feeder without divider (from left to right). All feeders were equipped with the SowMax ad-lib sow feed hopper, which has a rod that can be pushed sideways, opening a gap on the sides of the hopper to allow the feed to drop.

On approximately days 112 to 114 of gestation, sows were moved from the gestation facility into the farrowing house and randomly allotted to one of three lactation feeder types based on parity and caliper score (Knauer and Baitinger 2015), with sow serving as the experimental unit. Each of the three feeder types was equipped with the SowMax ad-lib sow feed hopper (SKU: 7150890500; Hog Slat; Newton Grove, NC). This feed hopper consisted of a rectangular metal box with a rod-like structure at the bottom of the hopper. This was installed on the farrowing stall headgate, with the bottom of the metal box matching approximately the top edge of the feeder bowl. For feed delivery, sows were required to push the rod from side to side, which moved internal parallel plates that allowed feed to drop from the feed hopper to the feeder bowl. When not triggered by the sows, the plates restricted the feed from flowing to the feeder bowls. The adjustment for the feed hopper was controlled by adjusting the distance between the plates. On the side of the metal box, there were six distance settings from 0 to 5, with 0 being fully closed, restricting all feed flow, and 5 allowing greatest feed flow (Rao et al., 2023). Feeder types consisted of 1) a dry lactation feeder with a separate nipple drinker available adjacent to the feeder; 2) a wet–dry lactation feeder with a divider to separate feed and water; or 3) the wet–dry lactation feeder without a divider (Figure 1). The wet–dry feeders had a nipple drinker located near the bottom of the feeder to allow sows free access to water. The three feeder types were placed in the farrowing stalls in the same sequence (Dry, wet–dry with divider, wet–dry without divider) from the front to the end of all farrowing rooms to balance and minimize any environmental effects in each room.

The same sorghum-soybean meal-based commercial lactation feed was fed to all sows (Table 1). Prefarrowing, sows were provided approximately 0.905 kg in the morning and afternoon, for a total of 1.81 kg per day, of the lactation diet. After farrowing, sows were provided ad libitum access to the lactation diet. The hopper of each feeder was filled twice a day, and each feed addition was weighed and electronically recorded. Feed addition to each feeder was registered to the stall with the date and weight recorded for calculating feed disappearance. Feeder adjustments were made daily to achieve approximately 50% feed coverage on the base of the feeder bowl. Feeder bowls were checked twice daily for wet or moldy feed, and this was removed when necessary. The spoiled feed was not weighed and, therefore, was counted as a portion of the total feed disappearance. Viable pigs were individually tagged with an RFID tag within 24 h of birth. The average weaning age was 20.9 d.

**Table 1.** Diet composition (as-fed basis)^1^

Item	Lactation diet
Ingredients, %	
Sorghum	64.21
Soybean meal, 46.5% CP	25.50
Corn distillers dried grains with solubles	5.00
Corn oil	1.50
Limestone	1.55
Monocalcium P (21% P)	0.70
Sodium chloride	0.55
Lysine, 60%^2^	0.44
Methionine hydroxy analogue^3^	0.08
L-Thr	0.08
Vitamin and trace mineral premix	0.25
Choline chloride	0.11
Phytase^4^	0.04
Total	100.00
Calculated analysis	
Standardized ileal digestibility AA, %	
Lys	1.05
Ile:Lys	68
Leu:Lys	148
Met:Lys	30
Met and Cys:Lys	53
Thr:Lys	64
Trp:Lys	20.0
Val:Lys	75
His:Lys	39
Total Lys, %	1.18
NE, kcal/kg	2,562
SID Lys:NE, g/Mcal	4.10
CP, %	19.4
Ca, %	0.80
P, %	0.54
STTD P, %	0.43

^1^Feed was manufactured by a commercial feed mill (JBS, Dalhart, TX).

^2^BioLys (Evonik Industries AG, Essen Germany).

^3^MHA (Novus International, Inc. Chesterfield, MO).

^4^OptiPhos Plus 2500 G (Huvepharma, Peachtree City, GA), provided 902 FTU/kg of diet for an estimated release of 0.11% STTD P.

### Data and Sample Collection

All animal and feed scales used in this trial were calibrated daily and verified with test weights to assure accuracy. All feed, sow, and litter data were recorded and stored using the LeeO system (Prairie Systems, Spencer, IA). An RFID tag was attached to each sow stall and identified as a location pen in the LeeO system. Individual information (sow ID, parity, and breeding date) for each sow was exported from the CloudFarms (Bratislava, Slovakia) system and then imported into the LeeO system. A walk-on platform scale was used to weigh sows before entering the farrowing house and at weaning. Sow caliper score was taken between days 109 and 111 of gestation (Knauer and Baitinger 2015). Caliper scores 6 to 12, 12 to 17, and 17 to 22 correspond to body condition scores of 1, 2, and 3, respectively. When sows were moved to the farrowing stall, they were also registered in the location pens in the LeeO system. Feed carts equipped with scales were used to obtain the weight of each feed addition. Feed additions were registered to the stall (location pen) with date and weight recorded for calculating total feed disappearance. Total feed disappearance was calculated by subtracting leftover feed in the feed hoppers at weaning from the cumulative feed additions during the lactation period. Total feed disappearance represents the combination of feed intake and feed wastage. The number of sows bred back by days 7, 14, and 30 were obtained from the CloudFarms system. Sows that were culled at weaning for any reason were not included in the subsequent farrowing data analysis. For litter performance, piglets were registered under the sow and location pen and were weighed individually at birth and at weaning. Nonviable piglets (under 0.68 kg body weight (**BW**) or dead before tagging), stillborn, and mummies were recorded but not included in the total litter weight. Any cross-fostering and mortalities throughout the lactation period were recorded.

After weaning, 3 farm employees were designated to wash feeders, and cleaning times were recorded. The number of feeders evaluated was 59, 57, and 57 for the dry feeder, wet–dry feeder with a divider, and wet–dry feeder without a divider, respectively. For economic data, the lactation diet cost was $363/tonne, the litter value was $1.54/kg of litter weight, and the labor cost for washing was $23/h.

### Statistical Analysis

For sow BW data, approximately 147 sows were used per treatment with an average parity of 1.9. Sows not included in the analysis of BW data were because of missing data points for their weaning weight. This was due to either culling or mortality prior to weaning. For feed disappearance, approximately 170 sows were used per treatment. Sows not included in the analysis of feed disappearance were a result of culling, mortality, or movement to another stall prior to weaning. For litter performance data, approximately 159 sows were used per treatment. Sows not included in the analysis of litter data were a result of missing data points for litter birth weight, weaning weight, culling, or mortality prior to weaning.

Data were analyzed using a generalized randomized block design for one-way ANOVA in the R Studio program. The lmer function from the lme4 package was used for lactating sow BW, sow caliper, feed disappearance, and litter growth performance. The glmer function (Poisson distribution) from the lme4 package was used for total born and litter size. The glmmTMB function (beta-binomial distribution) from the glmmTMB package was used for the percentage of mortality and pigs weaned. Sow (litter) was considered as the experimental unit. Farrowing room and group were the blocking factors for sow and litter data. The lactation feeder design was used as the fixed effect. Sow entry weight was used as a covariate for sow day 1 weights, weaning weight, and weight change data. The parity category (P1, P2, or P3+) was tested as a covariate for sow BW, feed intake, litter weights and growth performance, and economic variables and was included in the model when the Bayesian Information Criterion was decreased by at least 2.0. A Tukey’s/Sidak multiple comparison adjustment was used to control type I error rate. All results were considered significant at *P* ≤ 0.05 and marginally significant at 0.05 < *P* ≤ 0.10.

## RESULTS

There was no evidence of a difference in sow weight or caliper score on day 112 of gestation, at farrowing, or weaning, or their changes during lactation (Table 2). Total litter or pig birth weight, total pigs born, or pigs born alive as a percentage of total born, viable born, nonviable born, stillborn, or mummified pigs were not different amongst sows fed via the three lactation feeder designs. Litter or pig weaning weight or litter average daily gain were also not affected by lactation feeder design. However, sows fed via the dry lactation feeder had decreased (*P* < 0.05) total feed disappearance and average daily feed disappearance compared to those fed with either wet–dry feeder. As a result, litter feed efficiency was improved (*P* < 0.05) in sows fed with the dry feeder compared to those fed with the wet–dry feeders. In addition, sows fed with the dry feeder had decreased (*P* < 0.05) total lactation feed cost and feed cost per pig weaned compared to sows fed with either wet–dry feeder.

**Table 2.** The effect of lactation feeder design on sow and litter performance

	Feeder design				
	Dry	Wet–dry			
Divider:		Yes	No	SEM	*P*
Sow body weight, kg					
Sows, *n*	154	143	143		
Entry^2^	233.9	234.5	233.7	1.87	0.950
day 1^2^^,^^3^^,^^4^	196.6	197.0	196.0	0.91	0.739
Weaning^2^^,^^4^	195.2	197.7	196.6	1.35	0.310
Weight change					
Entry-weaning^2^^,^^4^	−38.8	−36.3	−37.4	1.35	0.310
Weight change, %^2^^,^^4^	−16.6	−15.2	−15.8	0.60	0.172
day 1 - weaning^2^^,^^3^^,^^4^	−1.2	0.7	0.7	1.63	0.550
Weight change, %^2^^,^^3^^,^^4^	−0.4	1.1	1.0	0.90	0.344
Sow caliper score					
Entry	14.6	14.7	14.7	0.18	0.889
Weaning	12.5	12.8	12.7	0.19	0.526
Change (entry to wean)	−2.0	−1.8	−2.1	0.21	0.500
Feed disappearance					
Sows, n	170	169	170		
Total feed disappearance, kg^2^	118.9^b^	128.4^a^	130.6^a^	3.41	< 0.001
Average daily feed disappearance, kg^2^	5.6^b^	6.1^a^	6.2^a^	0.10	< 0.001
Litter performance					
Sows, n	161	158	157		
Total born, n	16.6	16.6	16.8	0.34	0.852
Live born, %	91.3	91.0	90.8	0.66	0.827
Viable born, %	88.8	88.3	88.7	0.65	0.820
Nonviable born, %^5^	2.4	2.7	2.1	0.31	0.380
Stillborn, %	5.7	6.1	6.4	0.51	0.580
Mummified, %	3.0	2.9	2.8	0.34	0.914
Litter size after cross-fostering, n	14.8	14.8	14.8	0.31	0.979
Litter birth weight, kg^2^	21.11	20.81	20.98	0.435	0.754
Pig birth weight, kg^2^	1.42	1.42	1.42	0.037	0.937
Lactation length, d	21.0	20.8	20.8	0.57	0.894
Litter weaning weight, kg^2^	76.00	76.88	77.57	1.398	0.506
Pig weaning weight, kg^2^	5.90	5.90	5.90	0.096	0.995
Litter weight gain, kg^2^	54.88	56.07	56.59	1.341	0.369
Litter average daily gain, kg^2^	2.62	2.69	2.73	0.067	0.182
Litter feed efficiency^2^^,^^6^	0.474^a^	0.448^b^	0.445^b^	0.011	0.015
No. weaned	12.9	13.0	13.2	0.29	0.799
Weaned, %	86.8	88.5	88.9	0.81	0.135
Preweaned mortality, %^7^	13.2	11.5	11.1	0.81	0.135
Economics, $^8^					
Litter value^2^	117.3	118.6	119.7	2.16	0.506
Total lactation feed cost^2^	46.08^b^	49.92^a^	50.46^a^	1.25	<0.001
Litter value over lactation feed cost^2^	71.82	70.30	69.59	2.06	0.561
Feed cost per pig weaned^2^	3.63^b^	3.85^s^	3.89^a^	0.095	0.006
Feed cost per lb of litter weight gain^2^	0.39	0.41	0.41	0.010	0.112
Washing time per feeder, s	45.9^a^	40.0^b^	40.3^b^	4.67	0.001
Washing cost per feeder	0.28^a^	0.25^b^	0.25^b^	0.029	0.001
Sow subsequent performance					
Sows, n	157	151	139		
Bred by 7 d, %	73.2	74.3	78.7	6.99	0.524
Bred by 14 d, %	76.1	76.6	81.7	6.76	0.446
Bred by 30 d, %	85.1	85.4	89.0	4.09	0.534

^1^A total of 557 mixed parity sows (PIC 1050; average parity 1.9) that were bred to PIC 337 boars were used. Sow caliper score was taken between days 109 and 111 of gestation, and sows were allotted to treatment based on parity and caliper score.

^2^Parity category (P1, P2, or P3+) was used as a covariate.

^3^Day 1 weight estimated as: entry weight—(litter birth weight + estimated weight of conceptus). Conceptus weight was estimated using the equation proposed in NRC 2012. Weigh of conceptus (g) = exp (8.621 − 21.02 × exp (−0.053 × days of gestation) + 0.114 × total born) × Ratio.

Ratio = (total born × average piglet birth weight, g)/1.12 × exp {[9.095 − 17.69 exp (−0.0305 × 114) + 0.0878 × total born]}.

^4^Entry BW was used as a covariate.

^5^Nonviable pigs consisted of those born with an injury, a deformity, or under 0.68 kg BW.

^6^Litter feed efficiency = Lactation feed disappearance ÷ total litter weight gain.

^7^Preweaned mortality, % of litter size = [(dead after cross-fostering) ÷ (litter size at 24 h)] × 100%.

^8^Lactation feed cost was $330/ton, and the labor cost for washing was $23/h. Litter value = litter weaning weight × $1.54/kg.

^a,b^Means within a row with different superscripts differ (*P* ≤ 0.05).

There was no evidence for difference (*P* > 0.10) in subsequent reproductive performance (percentage bred by days 7, 14, and 30) between sows fed via the different feeder designs. Washing time and cost were greater (*P* < 0.05) for the dry feeders compared to both wet–dry feeder designs.

## DISCUSSION

Our hypothesis for initiating this study was based on the observations that growing-finishing pigs fed with a wet–dry feeder have greater growth rate and feed intake compared to those fed via a dry feeder (Nitikanchana et al., 2012; Myers et al., 2013; Bergstrom et al., 2014). The increased ADFI observed when using a wet–dry feeder is suggested to be a result of changes in feeding behavior, namely increased eating speed/reduced feeding duration resulting in the consumption of larger meals (Gonyou and Lou 2000; Hurst et al., 2008; Bergstrom et al., 2012b). We speculated that the same might be true during lactation. Rao et al. (2023) compared three ad libitum dry feeder designs on sow and litter performance. They observed that sows fed via the SowMax hopper with a curved feeder bowl had decreased feed disappearance and improved litter feed efficiency compared with those fed via an ad libitum PVC tube feeder also equipped with a curved feeder bowl. Therefore, we selected the SowMax hopper with a curved bowl and an adjacent waterer (as used by Rao et al. 2023) as our control feeder to compare wet–dry bowls that either divided water from feed or mixed feed and water.

The limited data on the effect of wet–dry feeders on lactation performance has shown positive results. However, many of these studies compared ad libitum-fed wet–dry feeders to hand- or limit-fed dry feeders and thus may not be applicable to modern sow farms that utilize ad libitum self-feeders in the farrowing house. Peng et al. (2007) observed that sows using a wet–dry feeder had increased feed disappearance and litter ADG. These improvements were suggested to be a result of sows having the ability to choose when and how much to eat and the ability to adjust the moisture level of each meal. However, as feed was only provided twice per day to sows using the conventional dry feeder, these sows may have had unintentionally limited feed availability. Thus, it is difficult to determine if feeder type, or feed accessibility, was the primary factor behind the improved performance. Pettigrew et al. (1985) also observed that sows fed using an ab libitum self-fed wet–dry feeder had increased feed disappearance and litter weight gain compared to sows fed by hand in conventional dry feeders. The hopper above feeders in our study allowed sows to have continual access to feed throughout the entire lactation period.

Due to labor limitations in this study, we were not able to separate actual feed intake from feed wastage, thus, data are presented as feed disappearance. In our study, the increased feed disappearance for sows using wet–dry feeders was not used by the sow because we observed no differences in litter growth rate or change in sow weight or caliper score. We believe that actual feed intake was similar between sows using all lactation feeder designs, but the greater disappearance was due to feed wastage for sows fed with wet–dry feeders. The proposed increase in feed waste when sows were fed using the wet–dry feeders led to a significant worsening of litter feed efficiency. The differences in feed wastage may have been due to excess water in the feeder bowl resulting in spilled feed, or due to greater quantities of spoiled feed removed from the wet–dry feeders by employees on a day-to-day basis. Spoiled feed was removed from feeders as it has been noted to increase feed refusal, thus reducing sow performance (Kanora and Maes, 2009). We believe this was largely due to the location of the nipple waterer within the wet–dry feeder, as feed particles may have gotten stuck in the nipple, causing excess water to collect in the bowl. From an economic perspective, the increased feed disappearance resulted in a 9% increase in total lactation feed cost for the wet–dry feeders compared to the dry feeder. As there were no improvements in litter growth or preweaning mortality, feed cost per pig weaned was increased (*P* = 0.006) for sows using the wet–dry feeders.

Another factor that must be considered when implementing a new feeder type into a commercial sow farm is the potential change in cleaning difficulty as this can affect the time required to clean feeders (Rao et al., 2023). Rao et al. (2023) observed a high amount of variability in cleaning times, largely due to variation from person to person, which reduced their ability to find differences in mean cleaning time between the different lactation feeders. We had a much lower degree of variability in our study and thus observed that the wet–dry feeders used in this experiment required significantly less time to clean compared to the dry feeder. This difference may be due to the differences in the shape of the feed pan between feeder types. As the dry feeder had a curved design to the bottom of the feed pan, a greater amount of time was required to successfully remove residual feed material as opposed to the flat design of the wet–dry feed pans.

In conclusion, the lack of differences in litter growth performance and sow weight change suggests that sows had similar true feed intake, regardless of feeder design. Therefore, the increased feed disappearance observed when sows were fed with either wet–dry feeder design used in this study appears to be due to increased feed wastage. As a result, sows using the dry feeder had improved litter feed efficiency and economic savings. Thus, transitioning existing sow farms to wet–dry feeders used in this study may not be economically justified.

## Abbreviations

ADFI: average daily feed intake
ADG: average daily gain
BW: body weight
CP: crude protein
DM: dry matter
G:F: , gain-to-feed
SID: standardized ileal digestibility
STTD: standard total tract digestibility
